# Assessing the mental health of slum dwellers: an ordinal logistic approach

**DOI:** 10.1186/s41043-024-00546-y

**Published:** 2024-06-03

**Authors:** Nasim Bin Jinnah Hijol, Nahid Salma, Indrani Sarker

**Affiliations:** Department of Statistics, Jahangirnagar University, Savar, Dhaka, 1342 Bangladesh

**Keywords:** Slum, Mental health, DASS-21, Khulna division, Bangladesh

## Abstract

**Background:**

Stress, depression and anxiety are prevalent mental health concerns that are getting worse every day in the context of rapidly expanding megacities, where a rising number of people live in slums. The purpose of this study is to evaluate the state of mental health and related variables underlying mental health issues among the impoverished population.

**Methodology:**

A total of 384 slum residents from the Khulna division responded to our questionnaire, which included the DASS-21 and other pertinent questions. Using ordinal logistic regression, the significant factors were extracted.

**Results:**

The Cronbach’s reliability coefficient for the DASS-21 scale lies between 0.79 and 0.89 which indicates the high reliability of the DASS-21 scales. According to the findings, roughly 72.7%, 84.1%, and 69% of participants slum dwellers experienced anxiety, depression, and anxiety problems respectively. The result of ordinal logistic regression shows, slum dwellers of female gender (B = 0.669*, 95% CI 0.141, 1.197), married (B = 1.506*, 95% CI 0.119, 2.893), having only one earning member in the family (B = 1.224*, 95% CI 0.526, 1.922), day laborers (B = 2.294*, 95% CI 1.115, 3.473), not being able to educate children due to financial problem (B = 0.558*, 95% CI 0.183, 0.932) were more likely to report high levels of anxiety, depression, and stress.

**Conclusion:**

The study finds that a significant portion of people who live in slums struggle with mental health issues. It also points to the need for further support, intervention, and study on Khulna's slum inhabitants who are experiencing mental health issues. The authors anticipate that the results will spur policymakers and government representatives to enhance financing for slum dwellers and employ psychological intervention strategies, both of which will aid in achieving the Sustainable Development Goal.

## Introduction

Our mental health is greatly impacted by several elements of our lives, including our social networks, spiritual practices, emotional health, and the environments in which we work and live [3]. There is growing concern about the impact that mental health issues are having on the worldwide burden of disease [27]. An increase in disability-adjusted life years is associated with neuropsychiatric diseases such as depression, substance misuse, and psychoses (DALYs). Clinically, depression is characterized by the following symptoms: forgetfulness, inattention, difficulty concentrating, ambivalence about making decisions, loss of interest in previously enjoyed activities (including sex), changes in eating habits (either bingeing or restricting), weight gain or loss, feelings of guilt, worthlessness, helplessness, restlessness, irritability, and even suicidal ideation [1]. Moreover, stress may be defined as a real or interpreted threat to the physiological or psychological integrity of an individual that results in physiological and/or behavioral responses [16]. Additionally, according to the American Psychiatric Association, anxiety is a feeling of unease, such as worry or fear, that can be mild or severe.

Slums are home to the disadvantaged worldwide. Slum communities are defined by squalor and very poor housing facilities. High living density, inadequate use of public resources, a lack of basic utilities, an unclean and polluted environment, low literacy rates, unemployment, crime, social, moral, and psychological deterioration, and poor health are the current conditions of slum area of Bangladesh [2, 23]. In [7] stated that only 5% of people in Bangladeshi slum communities have good-quality houses. In addition, many rural migrants cannot afford decent housing, they move into slum settlements, where they reside on vacant public or private land in densely populated communities. A recent analysis claims that slums of Bangladesh have population densities between 700 to 4210 persons per acre, with a minimum of four and a maximum of ten individuals sharing one room [5]. There, they remain susceptible to abrupt eviction by the government. Because they are seen as "illegal residents," those living in urban slum settlements are typically denied access to public sector resources, which significantly restricts their ability to get formal education and health care [20]. This overcrowding initiates inadequate sanitation and limited access to clean water, exacerbates the spread of infectious diseases, and contributes to physical health disparities [12]. According to [22], 60% of people in urban slums have access to safe drinking water but during floods, water sources become contaminated because slum areas are more likely to have broken or leaking pipes. In Bangladesh today, slums mostly offer an informal labor market that is marked by low pay, instability, and irregular work hours. Furthermore, a number of studies have documented the low mental health conditions of Bangladeshi slum dwellers, which they attribute to their precarious socioeconomic circumstances and the poor infrastructure of the slums in Bangladesh [10]. In addition, slum dwellers' mental health suffers from the ongoing stress of poverty, instability, and a lack of basic facilities, which raises their risk of developing anxiety, depression, and other psychological illnesses. These combined factors underscore the urgent need for holistic interventions addressing both the physical and mental health challenges within slum environments.

There are close to 2 million slum dwellers in Bangladesh, as reported by the Bangladesh Bureau of Statistics' most recent slum dweller and floating population census. The Dhaka division is home to 884,496 slum inhabitants, followed by Chittagong with 424,179, Khulna with 130,863, and Rajshahi with 65,526 [4]. There are 38,877 people living in slums in the Barisal division, 127,584 in Sylhet, and 92,470 in Rangpur, according to the report [4]. Among them, most of them are day laborers, rickshaw pullers, tailors, sweepers, domestic helpers, beggars, street peddlers, etc.

Unfortunately, there is very little high-quality research on the occurrence and progression of mental health problems in slum people in Bangladesh. Stress, depression, and anxiety are all too widespread in slum areas which leads to serious occurrences nowadays i.e., suicide taking the valuable lives of many slum people but they are not being appropriately addressed. Often, physical health concerns typically take precedence over the mental health difficulties of slum dwellers [15]. In the instance, it was shown that mental health problems had a major impact on slum life, including their ability to learn, find jobs, and engage in social activities. To prevent the loss of any precious lives owing to ignorance, a thorough inquiry is thus imperative in order to identify the causes of the decline in the mental health of slum dwellers and to take the appropriate corrective action. Thus, this study aims to evaluate mental health conditions and explore the contributing factors that lead to mental health problems in the slum population.

## Materials and methods

### Sample size

According to the Bangladesh Bureau of Statistics' most recent slum dweller and floating population census, 2022, the rough estimate of slum dwellers of Khulna is 1,30,863 [4]. This study was executed by collecting primary data from this population. Data was collected from the slum area of Khulna, Bangladesh between March 1, 2023 and April 1, 2023. Using Cochran's method, the necessary sample size was calculated with a 5% significance level and a 5% allowable margin of error (d = 0.05):

$${\text{n}}=\frac{{{\text{z}}}^{2}{\text{p}}(1-{\text{p}})}{{{\text{d}}}^{2}}$$

Therefore, 384 samples in total were required, which is why they were collected and taken into consideration for analysis.

### Study design

**Fig. 1:**
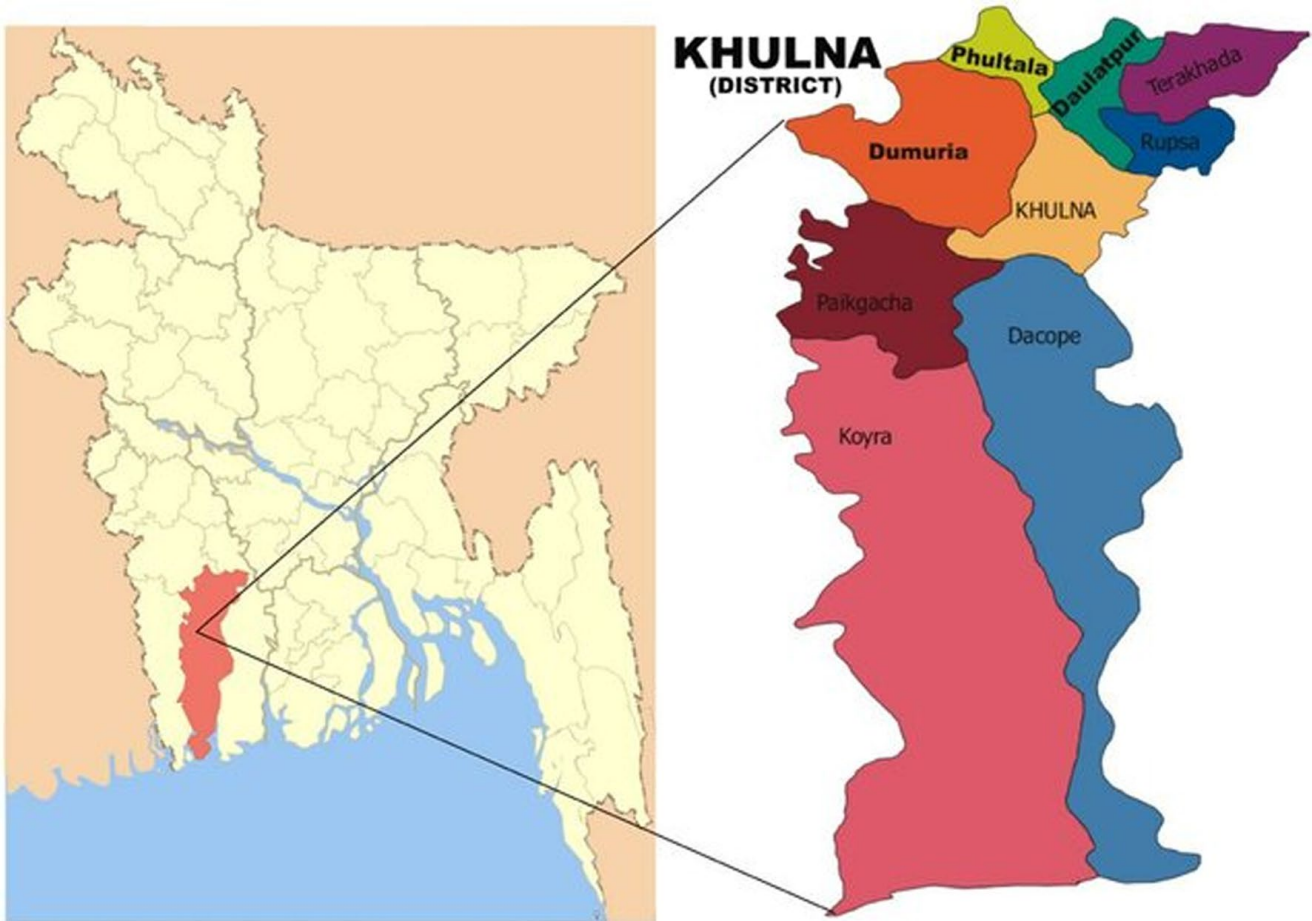
Location map of the study [8]

A cross-sectional study was carried out to evaluate the state of mental health and investigate the risk factors for mental health issues in the slum population. Given the time and cost constraints associated with conducting research in such circumstances, convenient sampling was employed to choose the sample from the population of interest due to its practicality and convenience of access to participants within slum communities. The questionnaires were distributed to easily accessible people of the Khulna slum area. Figure 1 displays the location of the study that was conducted. The purpose and importance of the research was explained to the participants. Also, before the survey ever being conducted, participants had received guarantees that their answers would remain confidential. To get the slum dwellers' verbal consent, the form has some initial Yes/No agreement questions. We had to read the self-administered questionnaire for the survey to the majority of the residents of the slums since they were illiterate.

### Questionnaire

The questionnaire was divided into two parts: socio-demographic information and mental health assessment.

#### Socio-demographic information

Socio-demographic information of the slum dwellers includes age range, gender, marital status, educational status, family size, occupation, number of earning members, monthly income, having serious patients in house, receiving government relief, and children going to school, having job permanency and experience food necessity.

#### Mental health assessment

The Depression, Anxiety, and Stress Scale-21 were used to assess the subjects' psychological well-being (DASS-21) [14, 17, 25]. The DASS-21 contains 21 questions, with 7 items dedicated to measuring stress, anxiety, and depression. Following data collection, participants' responses were numerically encoded using values between 0 and 3 (Never, Sometimes, Often, Almost always), and final scores were calculated by adding the values for each question

$${\text{Score}} = {\text{sum of rating points of each class}}*{2}$$

Total scores are represented as 0–9 = normal, 10–13 = mild, 14–20 = moderate, 21–27 = severe, and 28 +  = extremely severe [18].

### Statistical analysis

Descriptive statistics, such as frequency and percentage distributions of categorical data, have been used to characterize the participants. Cronbach's alpha was used to calculate the variables' reliability (ranging from 0 to 1). Since the response variables (anxiety, depression and stress) has more than two categories, the statistically significant variables were extracted using the ordinal logistic regression. The data was analyzed using IBM's SPSS (Social Science Statistical Software, Version 26.0).

## Results

### Mental health assessment

**Fig. 2:**
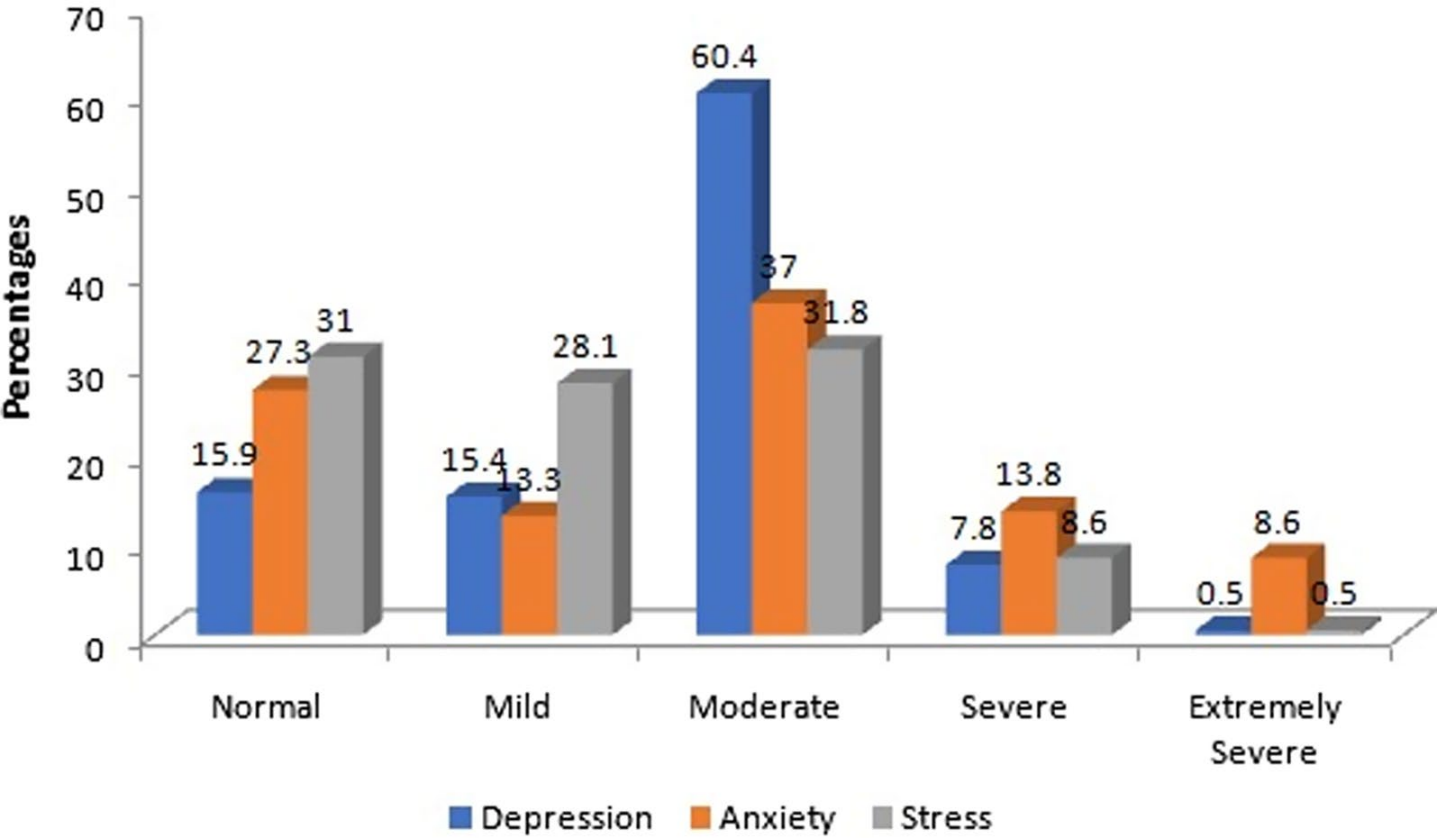
Participants' mental health status

Figure 1 displays the participants' mental health status. Among 384 participants, 60.4% are moderately, 15.4% are mildly, and 7.8% are severely depressed. Again, 37%, 13.3% 13.8%, and 8.6% are suffering from moderate, mild, severe, and extremely severe anxiety problems. Moreover, 31.8%, 28.1%, and 8.6% of slum people are struggling with moderate, mild and severe stress (Fig. 2).

### Association among anxiety, depression and stress scales

Table 1 represents the correlation between anxiety, depression, and Stress. Between the depression and anxiety scales, there is a high positive correlation (r = 0.747, *p* < 0.05). The association between anxiety and the stress scale is moderately positive (r = 0.648, *p* < 0.05). Additionally, stress and depression have a moderately positive connection (r = 0.662, *p* < 0.05). The findings indicate that all three of these measures are related, while depression and anxiety have a deeper connection.

**Table 1:** Correlation among anxiety, depression, and stress (N = 384)

Scales	Anxiety	Depression	Stress
Anxiety	1		
Depression	0.747	1	
Stress	0.642	0.662	1

### Socio-demographic & behavioral variable and mental health impact

The majority of slum residents are women (52.09%), people over the age of 50 (34.6%), and married (84.4%), as seen in Table 2, which also displays the association between other characteristics and mental health scales. Among the slum dwellers, most of them never go to school 150(39.1%), and found that most of them are day laborers 143(37.2%). Again, most of them claimed that they didn't get any relief from the government 239(62.2%) they experienced food necessity 260(67.7%) and their job is not permanent 224(58.3%).

**Table 2:** Frequency and percentage of socio-demographic variables of slums (N = 384)

Variable	Categories	Frequency (n)	Percentage (%)
Age range	25–35	104	27.1
	36–50	69	18
	Above 50	133	34.6
	Under 25	78	20.3
Gender	Female	203	52.9
	Male	181	47.1
Marital status	Married	324	84.4
	Unmarried	37	9.6
	Widowed	23	6
Education level	Higher level (Above 10 class)	15	3.9
	No schooling	138	35.9
	Primary level (0–5 class)	139	36.2
	Secondary level (6–10 class)	92	24
Family size	1–2	38	9.9
	2–5	239	62.2
	Above 5	107	27.9
Occupation	Day laborer	143	37.2
	Driver	20	5.2
	Factory worker	24	6.3
	Other	71	18.5
	Rickshaw puller	38	9.9
	Small business	71	18.5
	Tailor	17	4.4
Number of earning members in the family	1	302	78.6
	2 or Above	82	21.4
Family’s monthly income	10,000–15000 Taka	167	43.5
	5000–10000 Taka	109	28.4
	Above 15,000 Taka	80	20.8
	Under 5000 Taka	28	7.3
Having serious patients in the family	Yes	101	26.3
	No	283	73.7
Receiving relief from the government	Yes	145	37.8
	No	239	62.2
Children go to school	Yes	220	57.3
	No	164	42.7
Job permanency	Yes	160	41.7
	No	224	58.3
Experience food necessity	Yes	260	67.7
	No	124	32.3

However, the results of ordinal logistic regression analysis showed in Table 3 states that, slum dwellers between the age range 25–30 years had significantly lower scores for anxiety (B = − 1.718, 95% CI  − 2.6, − 0.835) and significantly higher scores for depression (B = 1.138, 95%CI 0.136,2.141). Again, female slum dwellers had significantly higher scores for anxiety (B = 0.669, 95% CI 0.141, 1.197) and stress (B = 0.969, 95% CI 0.422, 1.517). Meanwhile, both men and women were significantly associated with a lower score for anxiety (B = − 2.596, 95% CI  − 3.944, − 1.247) & (B = − 4.005, 95% CI  − 5.839, − 2.17) respectively and higher score for depression (B = 1.506, 95% CI 0.119, 2.893) & (B = 2.39, 95% CI 0.422, 4.368) respectively. Again, the slum dwellers with no schooling had a higher significant score for depression (B = 1.241, 95% CI 0.468, 2.014).

**Table 3:** Association among demographics characteristics and mental health impact of slum dwellers (N = 384)

Categories	n (%)	Anxiety B (95% CI)	Depression B (95% CI)	Stress B (95% CI)	
Age range					
25–35	104(27.1)	− 1.718*(− 2.6, − 0.835)	1.138*(0.136,2.141)	− 0.645(− 1.527,0.238)	
36–50	69(18.0)	0.236(− 0.745,1.218)	0.072(− 1.071,1.215)	− 0.151(− 1.137,0.835)	
Above 50	133(34.6)	− 0.522(− 1.535,0.491)	− 1.19(− 2.383,0.003)	0.473(− 0.556,1.501)	
Under 25	78(20.3)				
Gender					
Female	203(52.09)	0.669*(0.141,1.197)	− 0.395(− 0.994,0.204)		0.969*(0.422,1.517)
Male	181(47.1)				
Marital status					
Married	324(84.4)	− 2.596*(− 3.944, − 1.247)	1.506*(0.119,2.893)		0.573(− 0.642,1.789)
Unmarried	37(9.6)	− 4.005*(− 5.839, − 2.17)	2.395*(0.422,4.368)		0.214(− 1.53,1.958)
Widowed	23(6.0)				
Education level					
Higher level	15(3.9)	− 1.469*(− 2.764, − 0.174)	− 3.359*(− 4.66, − 1.851)		− 1.00(− 2.287,0.282)
No schooling	150(39.1)	0.021(− 0.634,0.676)	1.241*(0.468,2.014)		− 0.13(− 0.792,0.532)
Primary level	131(34.1)	1.617*(0.88,2.354)	0.59(− 0.246,1.427)		0.045(− 0.68,0.77)
Secondary level	88(22.9)				
Family size					
1–2	38(9.9)	0.314(− 0.589,1.217)	− 0.068(− 1.126,0.989)		− 1.385* (− 2.289, .48)
2–5	239(62.2)	− 0.438(− 0.995,0.118)	− 1.265*(− 1.919, − 0.61)		− 0.429(− 0.986,0.128)
Above 5	107(27.9)				
Occupation					
day laborer	143(37.2)	2.294*(1.115,3.473)	− 2.187*(− 3.464, − 0.91)		− 1.478*(− 2.65, − 0.31)
Driver	20(5.2)	2.014*(0.422,3.606)	− 4.439* (− 6.254, 2.62)		− 2.989*(− 4.6, − 1.378)
Factory worker	24(6.3)	0.95(− 0.531,2.432)	− 1.021(− 2.641,0.6)		− 1.204(− 2.672,0.264)
Rickshaw puller	38(9.9)	3.23*(1.896,4.565)	− 0.858(− 2.277,0.562)		− 0.849(− 2.148,0.45)
Small business	71(18.5)	1.161(− 0.138,2.461)	− 1.479*(− 2.91,0.048)		− 1.717*(− 3.02, − 0.41)
Tailor	17(4.4)				
Number of earning members					
1	302(78.6)	− 0.172(− 0.77,0.426)	1.224*(0.526,1.922)	1.394*(0.78,2.008)	
≥ 2	82(21.4)				
Monthly income					
10,000–15000	167(43.5)	0.74(− 0.486,1.965)	0.616(− 0.722,2.004)	− 0.433(− 1.65,0.784)	
5000–10000	109(28.4)	0.754(− 0.501,2.009)	1.186(− 0.251,2.623)	− 2.672*(− 3.946, − 1.4)	
> 15,000	80(20.8)	1.08(− 0.257,2.417)	1.44(− 0.089,2.97)	− 1.864*(− 3.208, − 0.52)	
< 5000	28(7.3)				
Having serious patients in the family					
No	283(73.7)	0.274*(− 0.149,0.696)	− 0.058*(0.518, 0.40)	− 0.302*(− 0.728, 0.12)	
Yes	101(26.3)				
Receiving relief from the government					
No	239(62.2)	− 0.351(− 0.731,0.029)	− 0.213(− 0.626,0.2)	0.173*(− 0.209,0.555)	
Yes	145(37.8)				
Children go to school					
No	16(42.7)	0.558*(0.183,0.932)	− 0.167(− 0.573, 0.24)	0.41*(0.034,0.786)	
Yes	220(57.3)				
Job permanency					
No	224(58.3)	− 0.318(− 0.696,0.06)	− 0.049(− 0.46,0.363)	0.082*(− 0.298,0.463)	
Yes	160(41.7)				
Experience food necessity					
No	124(32.3)	0.824*(0.427,1.221)	0.663*(0.234,1.092)	1.109*(0.703,1.515)	
Yes	260(67.7)				

On the other hand, who earn their livelihood by pulling rickshaws, being day laborers, and driving an auto were significantly associated with higher scores for anxiety (B = 3.23, 95% CI 1.896, 4.565), (B = 2.294, 95% CI 1.115,3.473) & (B = 2.014, 95% CI 0.422,3.606) respectively. The slum families having fewer earning members or one earning member were associated with positive significant scores for depression (B = 1.224, 95% CI 0.526, 1.922) and stress (B = 1.394, 95%: 0.78, 2.008). Slum families whose family income was between 5000 and 10,000 had lower significant scores for stress (B = − 2.672, 95% CI − 3.946, − 1.398). Moreover, the families who did not receive any government relief and have no job permanency are significantly associated with higher stress scores (B = 0.173, 95% CI  − 0.209, 0.555) and (B = 0.082, 95% CI  − 0.298, 0.463) respectively. The slum families having no serious patients in the house were significantly associated with higher anxiety scores (B = 0.274, 95% CI  − 0.149, 0.696) lower depression scores (B = − 0.058, 95% CI  − 0.518, 0.402), and stress score (B = − 0.302, 95% CI  − 0.728, 0.124).

## Discussions

Slum areas are a common phenomenon in Bangladesh as well as all over the world [13]. Complex socio-physical conditions found in slums can both endanger and benefit the mental health of their inhabitants [10]. Often, physical health concerns typically take precedence over the mental health difficulties of slum dwellers [15]. Therefore, this study aims to assess the mental health condition and the associated factors behind the mental health problems of slum people. A total of 384 slum residents' responses from the Khulna division were analyzed here.

It is clear from our study that the majority of respondents—roughly 34.6%—were over 50, with 52.9% of them being women and 47.1% being men. According to the findings, roughly 72.7% (mild = 13.3%, moderate = 37%, severe = 13.8% and extremely severe = 8.6%), 84.1% (mild = 15.4%, moderate = 60.4%, severe = 7.8% and extremely severe = 0.5%) and 69% (mild = 28.1%, moderate = 31.8%, severe = 8.6% and extremely severe = 0.5%) slum dwellers were suffering from anxiety, depression and stress respectively. This percentages are far higher than other Asian countries [24]. Unfortunately, there is a lack of information on the frequency of disease and the mental health of slum residents, which leads to inefficient distribution of health care resources and a failure to take preventative measures against disease [22]. Since mental health is linked to physical health, it is crucial to evaluate the conditions in urban slums where these poor people live [18].

As per the results of ordinal logistic regression, small families having 1–2 members have a lower significant effect on the stress of slum dwellers which is pretty obvious as the fewer the family members, the less the expenditure of money which leads to less stress. Furthermore, families in slums sometimes share a single room, with no separate space for the kitchen or latrine, and a lack of basic hygienic facilities [21]. It is pretty clear in our research that slum dwellers having no schooling (39.1%) have the highest significant depression score. Slum dwellers who have primary level education (34.1%) have a positive significant anxiety score. Among the slum dwellers, 37.2% were day laborers, 52% were drivers, and factory workers 6.3%, 9.9% were rickshaw pullers, 18.5% were small businesses, and 4.4% were tailors. Slum dwellers who earn their livelihood as day laborers and drivers have a higher anxiety score as they have limited income sources and the money, they earn is not enough to run their family which increases their anxiety level. It has also been reported [5] that, about 28% of people living in shantytowns relocate to urban areas in search of employment, about 50% do so due to financial hardship, and about 7% experience their homes and belongings washed away by rivers. Our findings also support previous studies [10] where they also found Social and physical factors (such as work satisfaction, capacity to generate revenue, population density, and environmental degradation) are associated with mental well being of Dhaka slums dwellers.

In addition, it is visible from our study that, around 78.6% of slum families have only one earning member which increases their depression and anxiety scores and a significant effect was found on that. This is obvious as the less earning member, has less ability to fulfill family's demands which will lead to more depression and stress. In General, most of the slum dwellers live on a daily income, which means they have little or no savings at all [21]. Moreover, around 5000–10000 and > 15,000 salary range slum dwellers were 28.4% and 20.8% respectively which are associated with lower significant stress scores due to having the necessary money to feed their family somehow. This finding strongly agrees with the outcome of previous study conducted by [18] where authors proved that financial condition is associated with mental well-being.

Recently the statistics of the International Labor Organization published that due to the economic crisis, most of the poor children drop out of school as poverty increases [19]. It has been found from our study that, around 42.7% of slum families can't send their children to school due to not having enough money which has higher significant anxiety and stress scores. Around 62.2% of slum dwellers received no government relief which has a higher significant stress score. In our study, around 67.7% of slum dwellers felt that food was a necessity. The emotional and mental toll of dealing with food poverty can be substantial, triggering or exacerbating a variety of preexisting conditions [6].

The poor mental health of Bangladeshi slum dwellers has severely disrupted their daily lives, underscoring the need for a more nuanced understanding of the importance of taking care of one's mental health [26]. However, the study revealed several causes for slum dwellers' mental health issues that should be taken into account for their betterment. In addition to offering sufficient government assistance, the government should look into a variety of online/offline seminars or programs that offer guidance to individuals on how to manage mental health concerns and reduce stress, anxiety, and depression. Residents of the informal settlements have been receiving food assistance from various organizations as part of various support initiatives implemented over the past few years [9, 11, 24]. The government and its legislators may put the study's conclusions into practice to guarantee that slum dwellers receive sufficient psychological compensation.

## Conclusion

This study allowed us to learn more about the state of mental health in the slums of Khulna, Bangladesh. The findings of the study demonstrated the serious mental health problems that residents of informal settlements face. The crucial elements influencing mental health are related to the female gender, being unmarried, having only one earning member in the family, day laborers were more likely to report high levels of anxiety, depression, and stress. Our findings point to the need for further support, intervention, and study on Khulna's slum inhabitants who are experiencing mental health issues. All pertinent factors should be considered when creating policies that will reduce the burden of mental illness by improving services for those in need and implementing early preventative measures. Organizations operating in slums must step forward for the development and supervision of slum people by providing different counseling services and financial help.

### Limitations

Convenience sampling may introduce selection bias since it only gathers data that is readily available, perhaps leaving out more difficult-to-find individuals. Because of this, it might not fairly reflect the entire population, which would restrict how broadly the results can be applied.

## Data Availability

On request, the data will be available at any time.
